# In this month’s Bulletin

**DOI:** 10.2471/BLT.19.000319

**Published:** 2019-03-01

In the editorial section, Linda-Gail Bekker et al. (170) examine governance of the HIV response in comparison to other global health programmes. Woranan Witthayapipopsakul et al. (171) announce an upcoming theme issue and call for papers on universal health coverage.

In the news section, Andrey Shukshin (174–175) reports on greater availability of cochlear implants in the Russian Federation. Paula Radcliffe explains to Gary Humphreys (176–177) why long-distance runners are particularly concerned about air pollution and introduces a clean air initiative of the International Associations of Athletics Federation.

## Bangladesh

### A decade of taxing cigarettes

Nigar Nargis et al. (221–229) find that tobacco taxes are insufficient to reduce consumption. 

## China

### Trying to eliminate viral hepatitis

Jue Liu et al. (230–238) describe progress in hepatitis B control.

## Fiji

### Minimizing damage from cyclones

Meru Sheel et al. (178–189) evaluate an early warning, alert and response system.

## Malawi, South Africa and United Republic of Tanzania

### Preventing mother-to-child transmission of HIV 

Harriet Jones et al. (200–212) study policy implementation.

## Thailand

### Investing tax revenue from tobacco and alcohol sales 

Suladda Pongutta et al. (213–220) convey lessons learned from a health promotion foundation.

## United States of America

### Counting deaths from unintentional injury

Peishan Ning (190–199) track changes in reporting methods.

## Global

### Designing control programmes

Seye Abimbola et al. (239–241) compare HIV to noncommunicable diseases.

### Improving infectious disease surveillance

Damien Ming et al. (242–244) propose harvesting data from connected diagnostic platforms.

